# Combination antimicrobial therapy: in vitro synergistic effect of anti-staphylococcal drug oxacillin with antimicrobial peptide nisin against *Staphylococcus epidermidis* clinical isolates and *Staphylococcus aureus* biofilms

**DOI:** 10.1186/s12941-024-00667-6

**Published:** 2024-01-20

**Authors:** Toktam Sharafi, Ezzat Allah Ghaemi, Maryam Rafiee, Abdollah Ardebili

**Affiliations:** 1 Infectious Disease Research Center, Faculty of Medicine, Golestan University of Medical Sciences, Gorgan, Iran; 2 Department of Microbiology, Faculty of Medicine, Golestan University of Medical Sciences, Gorgan, Iran

**Keywords:** Antimicrobial peptide, Biofilm inhibition, MRSA, MRSE, Nisin, *icaA*

## Abstract

The ability of *Staphylococcus epidermidis* and *S. aureus* to form strong biofilm on plastic devices makes them the major pathogens associated with device-related infections (DRIs). Biofilm-embedded bacteria are more resistant to antibiotics, making biofilm infections very difficult to effectively treat. Here, we evaluate the in vitro activities of anti-staphylococcal drug oxacillin and antimicrobial peptide nisin, alone and in combination, against methicillin-resistant *S. epidermidis* (MRSE) clinical isolates and the methicillin-resistant *S. aureus* ATCC 43,300. The minimum inhibitory concentrations (MIC) and minimum biofilm eradication concentrations (MBEC) of oxacillin and nisin were determined using the microbroth dilution method. The anti-biofilm activities of oxacillin and nisin, alone or in combination, were evaluated. In addition, the effects of antimicrobial agents on the expression of *icaA* gene were examined by quantitative real-time PCR. MIC values for oxacillin and nisin ranged 4–8 µg/mL and 64–128 µg/mL, respectively. Oxacillin and nisin reduced biofilm biomass in all bacteria in a dose-dependent manner and this inhibitory effect was enhanced with combinatorial treatment. MBEC ranges for oxacillin and nisin were 2048–8192 µg/mL and 2048–4096 µg/mL, respectively. The addition of nisin significantly decreased the oxacillin MBECs from 8- to 32-fold in all bacteria. At the 1× MIC and 1/2× MIC, both oxacillin and nisin decreased significantly the expression of *icaA* gene in comparison with untreated control. When two antimicrobial agents were combined at 1/2× MIC concentration, the expression of *icaA* were significantly lower than when were used alone. Nisin/conventional oxacillin combination showed considerable anti-biofilm effects, including inhibition of biofilm formation, eradication of mature biofilm, and down-regulation of biofilm-related genes, proposing its applications for treating or preventing staphylococcal biofilm-associated infections, including device-related infections.

**Supplementary Information:**

The online version contains supplementary material available at 10.1186/s12941-024-00667-6.

## Introduction

Biofilms are complex communities of bacteria attached to and embedded in a matrix composed of extracellular polymeric substances (EPS) [1]. The matrixes is mainly composed of exopolysaccharides, proteins, lipids, nucleic acids (eDNA and eRNA), and other biomolecules [1, 2]. EPS play major structural and functional roles that have crucial importance in the emergent properties of biofilms. EPS primarily strengthen microbial attachment to biological and abiotic surfaces. Then, further production of EPS forms a matrix that encloses and holds the cells firmly together, keeping them in close proximity and allowing intercellular interactions within a restricted space [1]. In addition, the EPS matrix also is a network that provides structural stability and functional environments that are essential for the biofilm lifestyle [3]. Likewise, EPS are responsible for enhanced tolerance or resistance of biofilm to antimicrobial agents and immune cells [2, 4].

Microorganisms within the biofilms can attach to both abiotic (e.g., almost all types of medical devices) and biotic (e.g., skin, bone, airway, connective tissue, intestinal mucosa, vascular endothelium) surfaces [5]. Therefore, biofilms may be associated with several tissue-associated chronic infections, in addition to their association with artificial surfaces [6]. Establishment of multilayered biofilm formation on medical devices results in device-related infections (DRIs) that are notoriously difficult to eradicate and often tend to relapse [7]. Under such condition, surgical removal and replacement of the device is often necessary and in cases where this is not a feasible option, patients require periodic antibiotic therapy for the remainder of their lives, causing a great morbidity and mortality [8, 9].

Although a wide range of bacterial and fungal species have been shown to cause biomedical device-related infections, *Staphylococcus epidermidis* and *S. aureus* are among the most common [10, 11]. Even, it has been suggested that *S. epidermidis* is responsible for nearly 80% of the bacteria causing medical DRIs [12]. Patients with prosthetic heart valves, cardiac devices, prosthetic joints, central lines, contact and intraocular lens, urinary and intravascular catheters, and intravenous drug use are at most risk of being infected with this member of the coagulase-negative staphylococci (CoNS) [13, 14]. A major clinical problem is that DRIs are often caused by methicillin-resistant *S. epidermidis* (MRSE), as well as multidrug-resistant (MDR) *S. epidermidis* and that the infections are naturally chronic due to formation of strong biofilm on the implanted devices, collectively hindering effective antibiotic therapy to clear infections [10, 15, 16]. The EPS molecule involved in biofilm formation in staphylococci has been named polysaccharide intracellular adhesin (PIA) based on function, or polyb-1-6-N-acetylglucosamine (PNAG) based on its chemical nature [17]. PIA, which facilitates cell to cell adhesion, is synthesized by the *ica* (intercellular adhesion) locus containing four different genes, *icaA*, *icaD*, *icaB*, and *icaC*. Expression of all four genes, which are arranged in an operon, is required for the synthesis of fully functional PIA [17]. The presence of the *icaADBC* gene family has been reported in *S. epidermidis* isolated from medical devices [18, 19].

Due to the complicated physical and biological properties of EPS matrix, biofilm-related infections are often not managed by conventional antimicrobial approaches, necessitating multi-targeted or combinatorial therapies. Therapeutic strategies that can generally be considered include preventing biofilm formation either by inhibiting the EPS production or blocking adhesin-mediated adherence and/or degrading the EPS in developed biofilms. As class I of bacterial-origin antimicrobial peptides (bacteriocins), lantibiotics or lanthionine-containing antibiotics, are promising therapeutic candidates exploring novel antimicrobial agents [20, 21]. Lantibiotics are ribosomally synthesized and post-translationally modified bio-active peptides (RiPPs) that have efficient bactericidal ability even against highly resistant superbugs, such as vancomycin-resistant enterococci (VRE) or methicillin-resistant *S. aureus* (MRSA), *Clostridioides difficile*, and some of them showed good activity in pre-clinical studies [22]. The lantibiotic nisin is the only bacteriocin legally approved as biopreservative and is used in the dairy industry to control contamination from *Listeria* strains [21]. Because of its wide-spectrum activity against both Gram-positive and Gram-negative pathogens, nisin is approved for clinical use as an alternative to antibiotics [21, 23]. Various studies have reported the applicability of nisin in the treatment of several infections, such as mastitis, oral, respiratory, and skin infections [24]. Nisin causes bacterial growth inhibition by pores formation in microbial cytoplasmic membrane (CM) and by interrupting the cell wall (CW) biosynthesis process through specific interaction with the precursor lipid II [23–25]. In a MRSA model, nisin was also shown to be associated with cell shrinkage and chromosomal DNA condensation, indicating that nisin interferes with DNA replication or segregation in *S. aureus* [26].

Previous study demonstrated high activity of nisin against both planktonic and sessile cells of several MRSA and *S. epidermidis* clinical isolates [27]. Furthermore, several studies have found synergistic effects through combination of nisin with various antimicrobials against both planktonic state and biofilms of different bacteria, including staphylococci [28–34]. The nisin-biogel has showed inhibitory capacity against *S. aureus* isolated from diabetic foot infections either in their planktonic and biofilm forms [35], and could be applied in combination with conventional antibiotics and antiseptics to improve their efficacy [36, 37]. With this in mind, the present study set out to evaluate the antibacterial activity of anti-staphylococcal drug oxacillin in combination with antimicrobial peptide nisin against clinical isolates of methicillin-resistant *S. epidermidis* (MRSE) and the standard strain methicillin (oxacillin)-resistant *S. aureus* ATCC 43,300 grown under routine culture conditions, biofilms, as well as biofilm-related gene *icaA*.

## Materials and methods

### Bacterial strains

The following bacterial strains were used in the present study: the *mecA* positive, methicillin (oxacillin)-resistant *S. aureus* (MRSA) (American Type Culture Collection ATCC^®^ 43,300^™^^*^), a reference strain originally isolated from United States, Kansas that was given as gift by Professor Mohammad Reza Pourshafie, Pasteur Institute of Iran, Tehran, and three clinical isolates of methicillin-resistant *S. epidermidis* (MRSE) that were recovered from clinical specimens by standard microbiological, biochemical, and molecular tests from a previous study [38] and then, the CLSI disk diffusion method with cefoxitin 30-µg disk (Rosco Diagnostica Co., Denmark) was used to identify methicillin resistance [39, 40]. *S. aureus* ATCC 43,300 was used as a control strain for detection of methicillin resistance, presence of the *icaA* gene, and biofilm production.

### Media

Brain heart infusion (BHI) broth (Merck KGaA, Darmstadt, Germany) was used for the cultivation of bacteria employed in the preparation of inocula for minimum inhibitory concentration (MIC) and genomic DNA extraction. Cation-adjusted Mueller-Hinton (CAMH) broth (Merck KGaA, Darmstadt, Germany) was used to determine the MIC, minimum biofilm-eliminating concentration (MBEC), and to perform checkerboard test. Tryptic soy broth supplemented with 1% glucose (TSB-glucose) (Condalab, Co, Madrid, Spain) was used for examination of biofilm formation and biofilm inhibition assays.

### Oxacillin and nisin preparation

Anti-staphylococcal antibiotic oxacillin sodium monohydrate (CAS#7240-38-2) and bacteriocin nisin from *Lactococcus lactis* (CAS#1414-45-5) were purchased from Sigma-Aldrich (Sigma-Aldrich Chemie GmbH,Taufkirchen, Germany). To prepare oxacillin stock solution, lyophilized oxacillin powder was dissolved in water. Nisin stock solution (10^6^ IU/g) was prepared by dissolving the lyophilized powder in hydrochloric acid (20 mM) to a concentration of 0.1 g/10 mL (10^4^ IU/g). All stock solutions were stored in a freezer (− 80 °C) until further use [39, 41].

### MIC determination

Antimicrobial activity of oxacillin and nisin against the planktonic cells was determined by the broth microdilution (BMD) method using CAMH broth according to the clinical and laboratory standards institute (CLSI) guidelines [39]. Briefly, each agent was serially diluted into a 96-well microtiter plate (JET Biofil, Guangzhou, China) at a volume of 100 µL of CAMH broth. The overnight bacterial culture of BHI broth was diluted to reach a density of approximately 1.5 × 10^8^ CFU/mL. The suspension was then diluted 1:20 to yield 5 × 10^6^ CFU/mL. A 10-µL aliquot of prepared suspension was inoculated to each well with different concentrations of antimicrobials, yielding the final test concentration of bacteria approximately 5 × 10^5^ CFU/mL. Antimicrobial activity was expressed as the MIC, the lowest concentration of each agent at which complete inhibition of bacterial growth is visually observed after 24 h of incubation at 37 °C. The experiments were performed in three independent biological replicates, each with three technical replicates.

### Biofilm formation assay

Biofilm formation was examined by crystal violet staining method as described previously [42]. An overnight culture of bacterial strains was adjusted to 0.5 McFarland, and then diluted 1:100 in TSB-glucose to yield a final concentration of approximately 1 × 10^6^ CFU/200 µL. A 200-µL aliquot was added to each well of a sterile microplate. Wells with TSB-glucose and inoculated suspension were considered negative and positive controls, respectively. After incubation at 37^˚^C for 24 h, contents of the wells were gently discarded and plates were washed three times with sterile phosphate-buffered saline (PBS, pH 7.3) to remove non-adherent bacteria. Adherent biofilm in each well was fixed with 99% methanol for 10 min, the solutions were removed, and the plate was dried. Biofilm in wells were stained with 200 µL 0.1% crystal violet (CV) (Merck KGaA, Darmstadt, Germany) for 5 min at room temperature, rinsed with water, and then dried. Biofilms were destained by treatment with 200 µL 95% ethanol for 30 min. Optical density (OD) of stained adherent cells was measured at 595 nm in a microtiter plate reader (BioTek, Bad Friedrichshall, Germany). Three biological replicates (each with two technical replicates) were carried out for all strains. A cut-off value (OD_cut_) as three standard deviations (SDs) above the mean OD of the negative control was established: OD_cut_ = average OD of negative control + (3 × SD of ODs of negative control). The following criteria were used for biofilm gradation in clinical isolates: non-biofilm-producer (-) if OD < OD_cut_, weak biofilm-producer (+) if OD_cut_ < OD < 2 × OD_cut_, moderate biofilm-producer (++) if 2 × OD_cut_ < OD < 4 × OD_cut_, and strong biofilm-producer (+++) if 4 × OD_cut_ < OD.

### Inhibition of biofilm formation assays

Activity of antibiotic or peptide alone. The ability of oxacillin or nisin to inhibit biofilm formation was investigated. Standard and clinical staphylococci were prepared at a concentration of 1 × 10^6^ CFU/200 µL in TSB-glucose from the overnight cultures. A 100-µL aliquot was added to the wells of a 96-well plate containing 100 µL of nisin or oxacillin alone at 1×, 1/2×, 1/4×, and 1/8× MIC. The wells containing inoculated bacterial strains without peptide or antibiotic were considered positive controls. After incubation at 37 °C for 24 h, contents of the wells were discarded, microplates were washed thrice with PBS, and then biofilm was stained with CV and OD_595_ was determined. The results expressed as the percentage of biofilm reduction compared with positive controls [41].

Combinatorial treatment of antibiotic and peptide. The effects of oxacillin and nisin in combinations against production of staphylococcal biofilms were evaluated by the BMD checkerboard technique with some modifications [41]. Briefly, serial dilutions of each of oxacillin and nisin were prepared and then mixed in four wells of the microplate in concentrations equivalent to 1×, 1/2×, 1/4×, and 1/8× MIC. A 100-µL aliquot of bacterial suspension at concentration of 1 × 10^6^ CFU/200 µL in TSB-glucose was added to each well containing both antibiotic and peptide. The positive controls were bacteria inoculated in TSB-glucose without antibiotic or peptide, and negative controls were medium with neither bacteria nor antimicrobial agents. After incubation, wells were rinsed three times with PBS, then the biofilm was stained with CV and OD was determined at 595 nm.

### Minimum biofilm elimination concentration (MBEC) assay

Susceptibility of *Staphylococcus* established biofilms was evaluated as previously described for MBEC assay [2]. The mature biofilm in a 96-well microtiter plate was washed thrice with PBS to remove planktonic cells. Antimicrobials were serially diluted to various concentrations ranging from 128 to 65,536 µg/mL for oxacillin and 32 to 16,384 µg/mL for nisin in CAMH broth. A 200 µL of each concentration was added in a corresponding well, and plates were incubated at 37ºC for 24 h. The well with established biofilm was used as the positive control and well containing CAMH broth with no peptide or antibiotic treatment was used as negative control. Then, contents of the wells were removed and wells were rinsed with sterile PBS to remove residual antimicrobials, and 200 µL of fresh CAMH broth was added to each well and allow to additionally incubate at 37 ºC for 24 h. The OD of wells was measured at 595 nm using a microplate reader. MBEC was defined as the minimum antimicrobial concentration that inhibited bacterial regrowth from the treated biofilm relative to the cell-only control.

### The effect of nisin on oxacillin MBEC

The combined effect of oxacillin and nisin on biofilms was evaluated as described previously described with some modifications [2, 43]. First, the 24 h biofilms were formed in 96-well microtiter plates and washed three times with PBS. Next, bacterial biofilms were challenged with different concentrations of oxacillin ranging from 4× to 2048× MIC and nisin at determined MBEC concentration. Following the overnight incubation, contents of the wells were removed. Then, microplate was washed with sterile PBS, after that, 200 µL of CAMH broth was added to wells for further 24-h incubation. Finally, MBEC of the oxacillin for biofilm cultures was determined as mentioned in “MBEC assay”.

### Polymerase chain reaction (PCR) assay and sequence analysis

Using the phenol-chloroform method, genomic DNA from the cells grown in a 24-h culture of BHI broth was extracted [40]. The presence of intercellular adhesion *icaA* gene was investigated in clinical isolates of *S. epidermidis* and *S. aureus* ATCC 43,300 by conventional PCR using the specific primers listed in Table 1 [44]. Each PCR mixture contained 12.5 µL Taq DNA Polymerase 2x Master Mix RED (Ampliqon, Odense, Denmark), including 1 × PCR buffer (Tris-HCl pH 8.5, [NH_4_]_2_SO_4_, 1.5 mM MgCl_2_, 0.2% Tween^®^ 20), 0.2 mM of each dNTPs, and Taq DNA polymerase 5 U/µL), 0.5 µL of 10 µM forward and reverse primers (0.2 µM), 1 µL of template DNA (5 ng), and sterile distilled water up to 25 µL. PCR was done in a Mastercycler gradient instrument (Eppendorf, Hamburg, Germany) with initial denaturation at 94 °C for 5 min, followed by 35 cycles of amplification (denaturation at 94 °C for 1 min, annealing at 56 °C for 1 min, and extension at 72 °C for 1 min), ending with a final extension at 72 °C for 5 min. The PCR products were electrophoresed on 2% agarose gel, visualized by DNA Safe Stain (SinaClon Bioscience Co., Tehran, Iran) and photographed under UV light. Sequencing of the PCR products was performed using reverse primer on the ABI by an ABI 3730xl DNA Analyzer (Applied Biosystem Inc., Forster City, CA, USA). The sequences were compared by the NCBI BLAST program (http://www.ncbi.nlm.nih.gov/BLAST/).

**Table 1:** Oligonucleotide primers used in cDNA synthesis and amplification by qPCR

Gene	Primer (5’→3’)	Amplicon size (bp)	T_m_ (°C)	Reference
*icaA* (for *S. epidermidis*)	F: TGCACTCAATGAGGGAATCA R: TAACTGCGCCTAATTTTGGATT	134	56	[44]
*16 S rRNA* (Reference gene)	F: GGGCTACACACGTGCTACAA R: GTACAAGACCCGGGAACGTA	176	56	[44]
*icaA* (for *S. aureus* ATCC 43,300)	F: ACACTTGCTGGCGCAGTCAA R: TCTGGAACCAACATCCAACA	188	56	[72]

### Gene expression analysis

Real-time quantitative reverse transcription (qRT) PCR was performed to determine the expression level of the *icaA* gene in staphylococci using the primers shown in Table 1. The *16 S rRNA* gene was used as an internal control to standardize expression levels between samples [45]. Staphylococcal cells were cultured in TSB-glucose and incubated at 37 ºC for about 4 h to reach mid-exponential phase. The standardized 0.5 MacFarland bacterial suspensions were diluted 1:100 in fresh TSB-glucose. These suspensions were then transferred into each well of a 12-well tissue culture microtiter plate. At this time, 1× MIC and 1/2× MIC oxacillin, nisin, and 1/2× MIC + 1/2× MIC combinations were added and incubated at 37 °C for 24 h. The wells containing bacterial suspension without peptide or antibiotic were used a controls. Wells were washed thrice with PBS, and then bacterial biofilms were harvested using the microprobe of an XL-2000 sonicator with sonication twice at amplitude 1.5 for 10 s with 1 min interval on ice (Qsonica LLC Co., Newtown, CT, USA) were harvested. The biofilm suspensions were centrifuged by centrifugation at 9,000× g 4ºC, for 10 min. The pellets were resuspended in 200 µL of lysostaphin-containing TE buffer (10 mM Tris HCl, 1 mM EDTA, pH 7.0) and incubated at 37 °C for 10 min. Total RNA from the pellets was extracted using the YTA Total RNA Purification Mini Kit (Favorgen Biotech. Corp., Kaohsiung, Taiwan), according to the manufacturer’s instruction. Extracted crude RNA was quantified spectrophotometrically (absorbance at 260 nm, A_260_), and treated enzymatically with RNase-free DNAse I (Fermentas, Thermo Fisher Scientific Inc., Vilnius, Lithuania) to remove contaminant genomic. RNA purity was measured by the absorbance ratio A_260_/A_280_. The quality of the purified RNA was also examined by 3% agarose gel electrophoresis. cDNAs was synthesized from 2.5 µg of DNAse-treated RNA samples using the AccuPower® RocketScript^™^ RT PreMix Kit (Bioneer, Republic of Korea) and 10 pM random hexamer (dN6) (Bioneer, Republic of Korea). The resulting cDNA was used as template in the real-time PCR on an ABI Prism^®^ 7300 instrument (Applied Biosystem Inc., Forster City, CA, USA) using the AccuPower^®^ 2X GreenStar^™^^*^ qPCR Master Mix (Bioneer, Republic of Korea). Amplification protocol included an initial denaturation step at 95 °C for 5 min, followed by 40 cycles of 95 °C for 20 s, 60 °C for 1 min, and 95 °C for 10 min. All three biological replicates were run in three technical replicates. The no reverse transcriptase control (NRT) samples were also run to check for genomic DNA contamination. The expression level of *icaA* gene was normalized between samples using *16 S rRNA* and calculated by the 2^–ΔΔCt^ method. A critical threshold cycle (CT) value was used to represent *icaA* transcript quantitatively. The ΔCTs for *icaA* transcript were calculated against that for the *16 S rRNA* reference gene. Results were obtained as the relative expression of *icaA* transcript in samples treated with nisin or oxacillin compared to that of non-treated controls.

### Statistical analyses

All experiments were performed in three biological replicates, each with three technical replicates and results were expressed as mean ± SD. Statistical analyses were performed using GraphPad Prism version 8.01 for Windows (GraphPad Software, La Jolla, California, USA). A one-way analysis of variance (ANOVA) followed by Tukey’s honest significant test was used to calculate and compare differences in the variables, including biofilm inhibition, MBEC, as well as the expression level of the *icaA* gene between the treated samples and control. All statistical analyses were done with a confidence level of 95%, and a *P*-value < 0.05 was considered statistically significant.

## Results

### Susceptibility

Cefoxitin disk testing showed the zone diameter of 0 for SE3, 19 mm for both SE1 and reference strain ATCC 43,300, and 20 mm for SE2, confirming resistance to methicillin (oxacillin). The result of in vitro activities of oxacillin and nisin against staphylococci studied are shown in Table 2. The reference strain *S. aureus* ATCC 43,300 had the accurate MIC value of 8 µg/mL described by CLSI [39]. The MRSE clinical isolates had MIC range from 4 to 8 µg/mL against oxacillin. In addition, the MIC values for nisin in reference strain and all but one clinical isolate (SE1, MIC = 128 µg/mL) were 64 µg/mL.

**Table 2:** In vitro anti-bacterial and anti-biofilm activities of oxacillin and nisin against staphylococci studied

^a^ Bacterial strain	Oxacillin			Nisin		
	MIC (µg/mL)	MBEC (µg/mL)	Fold change in MBEC/MIC ratio	MIC (µg/mL)	MBEC (µg/mL)	Fold change in MBEC/MIC ratio
SE1	4	8192	2048	128	2048	16
SE2	4	8192	2048	64	2048	32
SE3	8	2048	256	64	4096	64
SA ATCC 43,300	8	4096	512	64	4096	64

MBEC: Minimum biofilm eradication concentration, MIC: Minimum inhibitory concentration

^a^ SA ATCC 43,300 is a *mecA*-positive, methicillin (oxacillin)-resistant *Staphylococcus aureus* (MRSA) reference strain and SE1, SE2, and SE3 were three clinical isolates of methicillin-resistant *Staphylococcus epidermidis* (MRSE)

### Biofilm formation

The results of biofilm formation assay showed that all three MRSE isolates were categorized as strong biofilm-producer (+++), where the OD_595_ value corresponding to the amount of stained adherent cells ranged from 2.1 to 2.8) (*P* > 0.05). Similarly, the reference strain *S. aureus* ATCC 43,300 also produced a strong biofilm (OD_595_ = 2.7 ± 0.16).

### Inhibition of biofilm formation

**Fig. 1:**
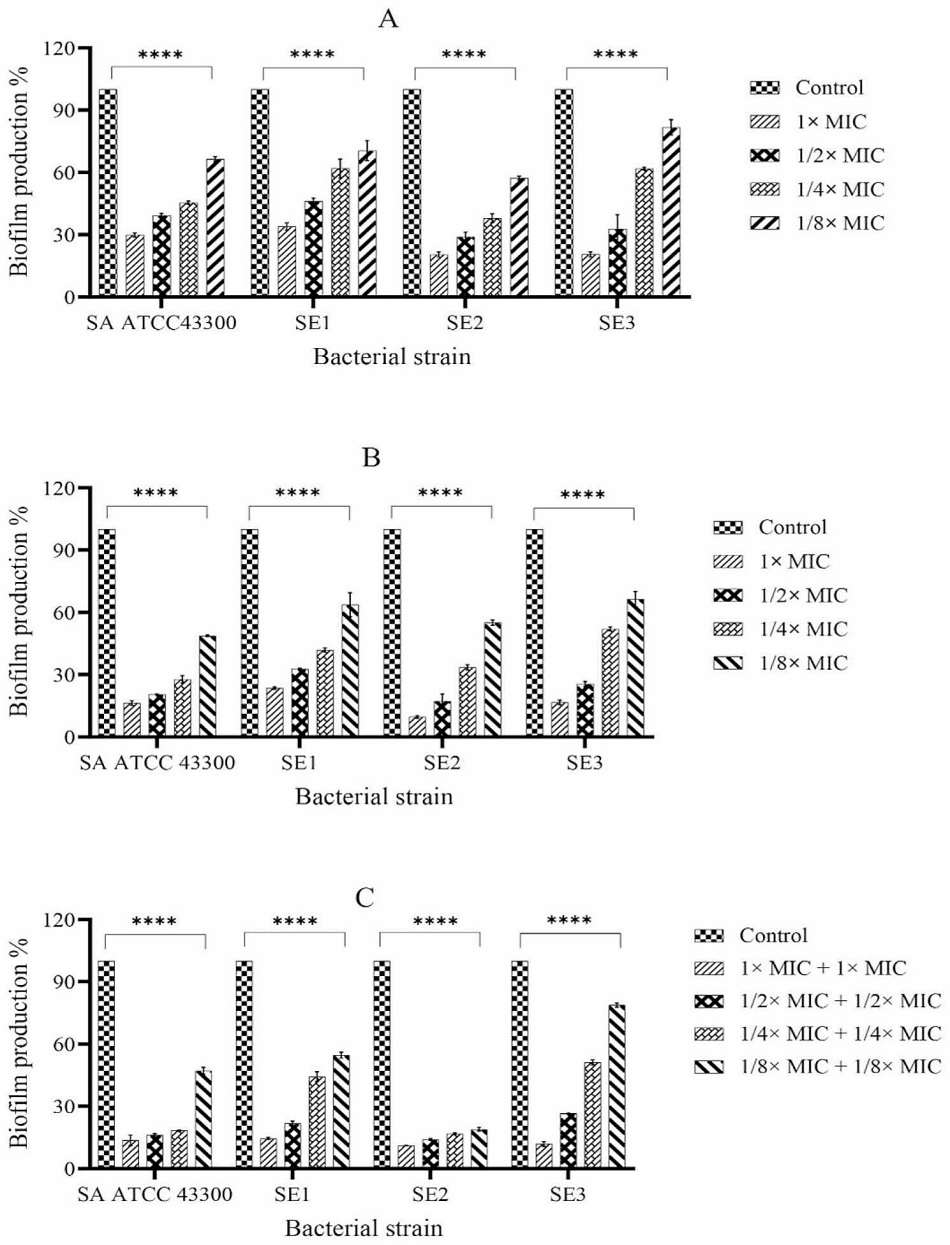
Effect of antimicrobial agents, alone (**A**, oxacillin and **B**, nisin) and in combination (**C**), on biofilm formation in staphylococci studied. The columns represent the average values of triplicate experiments and error bars represent the standard deviations. The asterisks represent the statistical difference between the groups and the control, determined by one-way analysis of variance (ANOVA) followed by Tukey’s honest significant test. Significance was accepted when the *P*-value was < 0.05 (****P < 0.0001). SA: *Staphylococcus aureus*, SE: *Staphylococcus epidermidis*, MIC: Minimum inhibitory concentration

For determining the effect of oxacillin and nisin alone on biofilm formation, we used the quantitative microtiter plate method. As shown in the Fig. 1A and B, both agents at all MIC concentrations (1×, 1/2×, 1/4×, and 1/8× MIC) showed significant inhibitory activity against biofilm formation at 24 h compared to cells incubated in medium only (*P* < 0.0001). In addition, this effect was concentration-dependent in all groups (*P* < 0.0001). Higher doses of antimicrobial exposure remarkably prevented biofilm development (up to 79.5%±1.25 and 90.36%±0.57 by oxacillin and nisin at 1× MIC, respectively, both in SE2), whereas lower antimicrobial doses caused less biofilm inhibition (up to 18.21%±3.7 and 33.52%±3.61 by oxacillin and nisin at 1/8× MIC, respectively, both in SE3).

### Combination effect of oxacillin and nisin on biofilm formation

Combination of nisin and oxacillin at four concentrations, starting from 1× MIC were tested by a checkerboard manner. Results showed biofilm biomass reduction was significantly enhanced by combinations of antimicrobials at 24 h in relation to concentrations compared to when each antimicrobial was used alone at the same concentrations (Fig. 1C). Particularly, 1/4× MIC + 1/4× MIC and 1/8× MIC + 1/8× MIC combinations considerably inhibited biofilm formation in SE2 isolate than the use of same concentrations of agents alone (83.17%±0.41 and 81.01%±0.81 reduction in biofilm, respectively) (Fig. 1C).

### MBEC values

The results showed that all staphylococci increased considerably their resistance to both agents. Oxacillin and nisin eradicated all MRSE isolates with MBEC values ranging from 2048 to 8192 µg/mL and 2048 to 4096 µg/mL, respectively. Both agents eradicated MRSA reference strain with MBEC value of 4096 µg/mL (Table 2).

### The effect of nisin on oxacillin MBEC

Addition of nisin at distinct MBEC concentration ranging from 2048 µg/mL to 8192 µg/mL significantly decreased the oxacillin MBECs from 16- to 32-fold in MRSE and 8-fold in ATCC 43,300 reference strain, indicating the synergistic effects between the AMP and antibiotic (Table 3).

**Table 3:** The combined effects of oxacillin and nisin on MBEC values of oxacillin in staphylococci studied

^a^ Bacteria	MBEC value (µg/mL) of		Fold reduction in oxacillin MBEC in the presence of nisin
	Oxacillin	Oxacillin + nisin	
SE1	8192	256	32
SE2	8192	512	16
SE3	2048	128	16
SA ATCC 43,300	4096	512	8

MBEC: Minimum biofilm eradication concentration

^a^ SA ATCC 43,300 is a *mecA*-positive, methicillin (oxacillin)-resistant *Staphylococci aureus* (MRSA) reference strain and SE1, SE2, and SE3 were three clinical isolates of methicillin-resistant *Staphylococcus epidermidis* (MRSE)

### PCR-sequencing analysis

All staphylococci studied carried chromosomal *icaA* gene in PCR analysis. The 188 bp- and 134 bp-band size of amplicons were detected for the *icaA* gene in *S. aureus* ATCC 43,300 and *S. epidermidis* clinical isolates, respectively (Supplementary Information file). Sequencing data confirmed the presence of the *icaA* gene in clinical isolates and reference strain.

### The effect of oxacillin and nisin on the expression of *icaA* gene

**Fig. 2:**
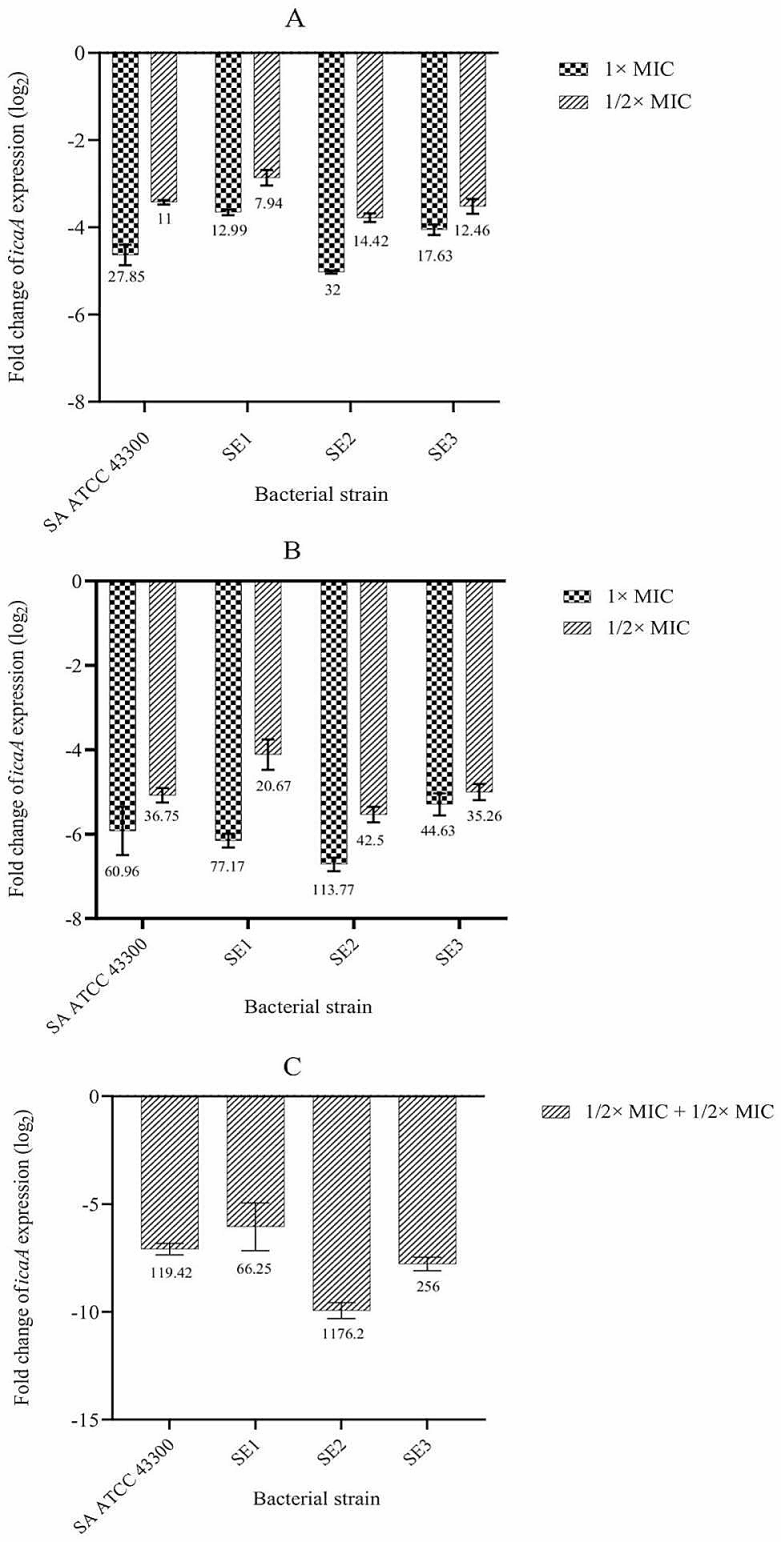
Real-time quantitative PCR analysis of the *icaA* gene transcription in staphylococcal biofilms incubated with antimicrobial agents, alone (**A**, oxacillin and **B**, nisin) and in combination (**C**). The expression level of *icaA* was normalized to the *16 S rRNA* gene. The columns represent the average values of triplicate experiments and error bars represent the standard deviations. The one-way analysis of variance (ANOVA) followed by Tukey’s honest test indicated a significant difference between each of the strains and untreated groups. Significance was accepted when the *P*-value was < 0.05. SA: *Staphylococcus aureus*, SE: *Staphylococcus epidermidis*, MIC: Minimum inhibitory concentration

The expression level of *icaA* gene in staphylococci incubated with the oxacillin and nisin is shown in Fig. 2. The level of expression was significantly decreased during treatment with 1× MIC and 1/2× MIC of each agent alone compared with the control groups (*P* < 0.0001) (Fig. 2A and B). Treatment with oxacillin down-regulated the gene ranging from 12.99-fold (in SE1) to 32-fold (in SE2) at 1× MIC concentration and 7.94-fold (in SE1) to14.42-fold (in SE2) at 1/2× MIC concentration, while the gene was down-regulated ranging from 44.63-fold (in SE3) to 113.77-fold (in SE2) and 20.67-fold (in SE1) to 42.5-fold (in SE2) during treatment with nisin at 1× MIC and 1/2× MIC concentrations, respectively. Notably, the reduction of *icaA* expression was significantly higher with 1/2× MIC combinatorial treatments than with oxacillin or nisin alone (*P* < 0.0001) (Fig. 2C).

### Nucleotide sequence accession number

The sequences of staphylococcal *icaA* gene has been submitted to NCBI and deposited in the GenBank database under the accession numbers OR752439 and OR752440.

## Discussion

Generally, treatment of biofilm-related infections is very difficult due to several factors, such as the reduction of drug penetration and release by the extracellular matrix, the slow growth rate of cells in the biofilm, and the presence of silent cells [34, 46]. To overcome the biofilm-associated resistance, novel therapeutic strategies have been today of interest to develop effective antimicrobial agents against these infections [41].

Various studies have demonstrated the ability of AMPs to inhibit biofilm formation or degrade of mature bacterial biofilms [47–49]. In recent years, application of AMPs in combination with conventional antibiotics has been shown to be effective against the biofilm structures as a viable therapeutic approach [15, 41, 50, 51]. It also allows reducing the dosages, attenuating the rates of adverse events, and enhancing the selective toxicity of antibiotics [52, 53]. Here, we examined the effects of combining the prototypical lantibiotic nisin and conventional antibiotic oxacillin on biofilm formation and/or eradication of *S. aureus* ATCC 43,300 and *S. epidermidis* clinical isolates. Furthermore, we revealed that the combinations were more effective in reducing the expression level of *icaA* gene during biofilm formation compared to when either antimicrobial is used alone.

In this study, we first evaluated the in vitro activities of antimicrobials alone against standard MRSA and clinical MRSE planktonic cells. The MICs for oxacillin and nisin ranged from 4 to 8 µg/mL and 64 to 128 µg/mL, respectively. When we assessed the anti-biofilm activities of these antimicrobial agents, the MBEC values for oxacillin and nisin ranged between 2048 and 8192 µg/mL and 2048 to 4096 µg/mL, respectively, indicating that mature biofilms are highly resistant to antimicrobial agents [54, 55]. Under such conditions, the dosage regimens of the clinically used antibiotics that have primly developed for treatment of infections due to the planktonic bacteria are ineffective to eradicate their biofilms. The MBEC/MIC ratio of oxacillin and nisin in the present study ranged from 256- to 2048-fold and 16- to 64-fold, respectively, that is in line with findings obtained by studies working on different antibiotics and AMPs [15, 41, 56]. The MBEC/MIC ratio is one of the important factors for choosing the antibacterial agents in the treatment of biofilm-related infections. Although the MIC values of nisin were higher than those of oxacillin, it is noteworthy that the MBEC/MIC ratio of nisin was significantly lower than that of oxacillin (*P* < 0.05), suggesting the higher anti-biofilm effect of nisin compared to oxailiin. Okuda et al., have revealed that nisin A is significantly effective against MRSA and other staphylococcal biofilms. They found that 4× MIC concentration of nisin A killed completely *S. aureus* MR23 during 1-h incubation. Nisin A showed also high activity against *S. aureus* MR23 biofilm as time- and dose-dependent manner. In addition, after treatment of other staphylococcal biofilms, including that of *S. epidermidis* with various bacteriocins at a concentration of 4× MIC for 1 h, nisin A showed the highest activity, with the majority of dead cells constituting the biofilm. Their further experiments indicate that nisin A and other pore-forming bacteriocins might be effective for the prevention and treatment of biofilm-associated infections in clinical applications [27].

Our results showed that both oxacillin and nisin significantly inhibit biofilm formation in a dose-dependent manner (*P* < 0.0001). Notably, the maximum impact on biofilm formation was found for SE2, where 90.36% and 79.5% of 24-h biofilm was inhibited by nisin and oxacillin, respectively, at growth inhibitory concentration. Similarly, the study by Qu et al., demonstrated that biofilm of some CoNS is increased by oxacillin in a concentration-dependent manner [57]. Wang et al., showed that single oxacillin treatment at 1/2× MIC inhibited the biofilm formation in 2 out of 4 MRSA strains, while significantly stimulated on the two other MRSA studied, especially on the methicillin-sensitive *S. aureus* (MSSA) ATCC 25,923 [58]. In addition, they found that the biofilm producing in all but one MRSA strains decreased compared with control after treated with nisin at same concentration [58]. Although we didn’t observe in the present study, inducing biofilm formation by oxacillin or nisin has been reported by previous studies [59–62]. Mirani et al., found that exposure to sub-MICs of methicillin led to a considerable increase in biofilm production in *S. aureus* USA300 and USA500 that was mediated by autolysis activity of *atl* [62]. This indicates that a genetic mechanism causes bacterial lysis to liberate eDNA that can enhance biofilm production [63]. Likewise, Sudagidan and Yemenicioğlu observed that nisin at MIC concentration (25 µg/mL) reduced or inhibited biofilm formation in all *S. aureus* strains, but some of them continued to form biofilm at sub-inhibitory concentrations [64]. These studies suggest a strain-dependent resistance among *Staphylococcus* spp. to oxacillin and other related compounds, as well as nisin.

In the present study, oxacillin + nisin treatment could effectively inhibit biofilm formation compared to single treatments. Mataraci and Dosler demonstrated that treatment of MRSA ATCC 43,300 biofilms in vitro with nisin improves the efficacy of daptomycin, linezolid, teicoplanin, azithromycin, and ciprofloxacin in bacterial killing than antibiotic treatment alone [41]. The results of study by Field et al., revealed that sub-inhibitory levels (1/5× MIC and 1/4× MIC for colistin and nisin, respectively) can effectively prevent biofilm formation in *Pseudomonas aeruginosa* PAO1 through total inhibition of growth, thereby enhancing efficacy, and ultimately, restoring sensitivity [53]. Beta-lactam antibiotics (e.g., oxacillin) target bacterial CW biosynthesis. When the bacteria are exposed to oxacillin, bacterial wall morphology changes, whereas in combination with nisin, morphological changes also occur in both bacterial CM and CW that facilitates nisin penetration into the bacterial cell [65, 66]. This mechanism explains how AMPs act in synergy with conventional antibiotics against planktonic cells. But, what about for biofilms? The physiochemical properties of non-living surface, such as hydrophobicity, roughness, and a predisposition to protein adsorption play generally an important role in attachment of microorganisms to surfaces and the subsequent biofilm development [67]. Furthermore, adhesion is thermodynamically considered favorable only if the process results in a decrease in total free energy [68]. Pimentel-Filho et al., have showed that bacteriocins (e.g., nisin and bovicin HC5) change the hydrophobicity of polystyrene surfaces, causing decrease in bacterial attachment. Since the total free energy of adhesion between the surface and the bacterial cell is positive, in the medium containing bacteriocins, the adhesion process is considered unfavorable, indicating the second reason for biofilm inhibition [68]. Collectively, these findings highlight that combinations of antimicrobial agents have greater potential than single treatments to prevent biofilm formation, and at the same time suggesting a potentially synergistic effect of nisin with oxacillin.

Considering the important role of *icaA* gene in PIA synthesis and biofilm development in staphylococci [69], we assessed its transcription level as an index of biofilm formation by real-time PCR. Our results indicated that the expression was down-regulated when staphylococal biofilms were treated by either nisin or oxacillin alone at both 1× MIC and 1/2× MIC concentrations compared to controls (*P* < 0.0001), supporting the microtiter plate method findings. These results are in agreement with those reported by Zhu et al., who found that human β-defensin 3 (HβD3) significantly decreased as dose-dependent manner the expression of both *icaA* and *icaD* genes in MRSE ATCC 35,984 [70]. Similarly, Saising et al., demonstrated that gallidermin inhibits not only the growth of staphylococci in a dose-dependent manner but also effectively prevents biofilm formation by both *S. aureus* and *S. epidermidis*. They found that the effect of gallidermin on biofilm was due to repression of biofilm-related genes *icaA* and *atlA* (major autolysin). These data imply that biofilm inhibition depend on reduced PNAG synthesis, a significant component of the staphylococcal biofilm matrix [48]. In contrast, Mirzaie et al., reported that expression of *icaA* and *atlE* genes were up-regulated in *S. epidermidis* against sub-MIC concentrations of cloxacillin, cefazolin, and clindamycin, suggesting antibiotic-induced biofilm development. However, vancomycin was able to down-regulate *icaA* and *atlE* [60]. Notably, we found that the reduction of *icaA* expression was significantly more pronounced when the bacteria were exposed to the combination of two antimicrobial agents at 1/2× MIC concentrations (*P* < 0.0001). Minich et al., found that oxacillin at the 1/2× MIC concentration decreased the relative mRNA expression of *icaA* in the biofilm-producing strain *S. epidermidis* RP62A comparison to untreated control (*p* < 0.01). In the presence of oxacillin (1/2× MIC) and vanillin (1/20× MIC), *icaA* expression decreased by 55% (*P* < 0.0001), highlighting the advantages of combinatorial strategy in repressing the biofilm determinant genes [71].

## Conclusions

The data presented here demonstrates the potential for nisin and conventional antibiotic combinations to act as potent antimicrobial and anti-biofilm agents against MDR pathogens, including *S. epidermidis* and *S. aureus* which form biofilm on in-dwelling devices or hospital equipment and have been shown to be the most common pathogens associated with DRIs. The enhanced anti-biofilm activity of nisin/oxacillin combinations found here against staphylococci suggests their future applications as novel approach to eliminate problematic biofilms and associated infections. It is expected that future researches will provide vital new information towards the understanding all aspects of this new strategy in the clinical applications.

### Electronic supplementary material

Below is the link to the electronic supplementary material.

Supplementary Material 1

## Data Availability

The datasets generated and analyzed during this research were included in the main document of this manuscript.

## Abbreviations

ANOVA: One-way analysis of variance
ATCC: American Type Culture Collection
BMD: Broth microdilution
BHI: Brain heart infusion
CAMH broth: Cation-adjusted Mueller-Hinton broth
CLSI: Clinical and laboratory standards institute
CM: Cytoplasmic membrane
CoNS: Coagulase-negative staphylococci
CT: Critical threshold cycle
CV: Crystal violet
CW: Cell wall
DRIs: Device-related infections
EPS: Extracellular polymeric substances
HβD3: Human β-defensin 3
*ica*: Intercellular adhesion
MBEC: Minimum biofilm-eliminating concentration
MDR: Multidrug-resistant
MIC: Minimum inhibitory concentration
MRSA: Methicillin-resistant *S. aureus*
MRSE: Methicillin-resistant *S. epidermidis*
NRT: No reverse transcriptase control
OD: Optical density
OD_cut_: Optical density cut-off value
PBS: Phosphate-buffered saline
PCR: Polymerase chain reaction
PIA: Polysaccharide intracellular adhesion
PNAG: Polyb-1-6-N-acetylglucosamine
dN6: Random hexamer
RiPPs: Ribosomally synthesized and post-translationally modified bio-active peptides
qRT PCR: Real-time quantitative reverse transcription PCR
TSB-glucose: Tryptic soy broth with 1% glucose
VRE: Vancomycin-resistant enterococci
