# Supplementary figures and images for: Combination antimicrobial therapy: in vitro synergistic effect of anti-staphylococcal drug oxacillin with antimicrobial peptide nisin against Staphylococcus epidermidis clinical isolates and Staphylococcus aureus biofilms
Source: Ann Clin Microbiol Antimicrob. 2024 Jan 20;23:7. doi: 10.1186/s12941-024-00667-6 (PMC10800071; doi:10.1186/s12941-024-00667-6)

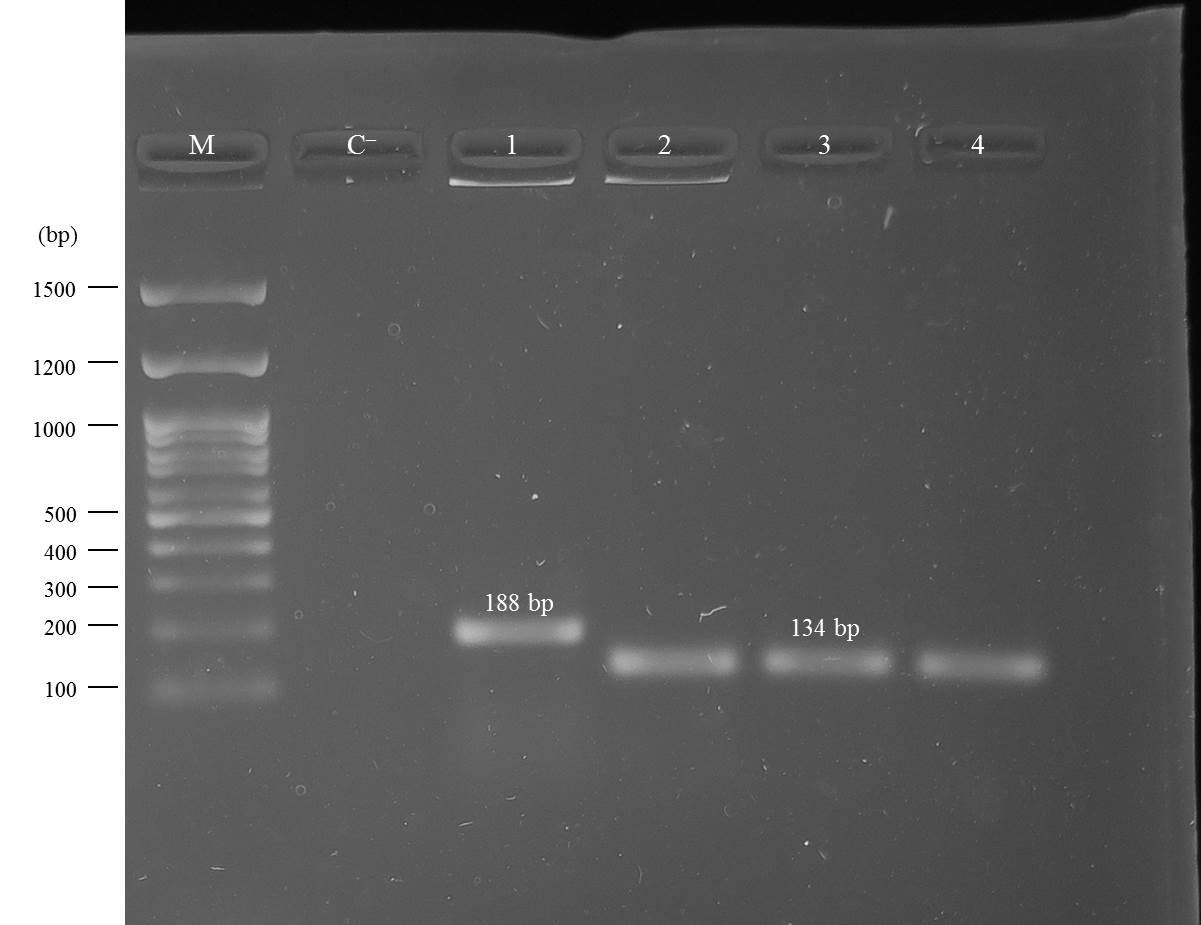

Supplement: Supplementary file 1 — Supplementary Material 1 [file 12941_2024_667_MOESM1_ESM.jpg]
